# Wherefrom and whereabouts of an alien: the American liver fluke *Fascioloides magna* in Austria: an overview

**DOI:** 10.1007/s00508-014-0499-3

**Published:** 2014-02-18

**Authors:** Helmut Sattmann, Christoph Hörweg, Larissa Gaub, Anna Sophia Feix, Michaela Haider, Julia Walochnik, Wolfgang Rabitsch, Heinrich Prosl

**Affiliations:** 1 3. Zoology (Invertebrates), Natural History Museum Vienna, Burgring 7, 1010 Vienna, Austria; 2 Institute of Specific Prophylaxis and Tropical Medicine, Medical University Vienna, 1090 Vienna, Austria; 3 Center for Advanced Bioanalysis GmbH, 4020 Linz, Austria; 4 Department Biodiversity and Nature Conservation, Environment Agency Austria, 1090 Vienna, Austria; 5 Department of Pathobiology, Institute of Parasitology, Vetmeduni Vienna, 1210 Vienna, Austria

**Keywords:** Digenea, Trematoda, Invader, Dispersal, Austria, Digenea, Trematoda, Neobiota, Ausbreitung, Österreich

## Abstract

The giant liver fluke *Fascioloides magna*, an invasive species originating from North America, was recorded in Austria in the wild for the first time in 2000. Since then, various data concerning the epidemiology in snail intermediate hosts and cervid final hosts have been reported. *Galba truncatula* acts as snail intermediate host, and red deer, roe deer and fallow deer act as final hosts. *G. truncatula* is abundant throughout the region, especially along muddy shores of slow-flowing branches of the river system. Prevalence in deer (20–100 %) is much higher than in snails (0.03–0.2 %). Despite medical treatment of parts of the deer population, the parasite has successfully established itself on both sides of the Danube floodplain environments southeast of Vienna. Genetic analysis revealed that the infection of Austrian deer populations apparently originated from foci in the Czech Republic or from populations of Danube tributaries. Areas adjacent southwards, which will soon be joined through wildlife crossings, have not yet evidenced *F. magna*. Nonetheless, these environments are inhabited by host snails and deer and therefore constitute suitable habitats for *F. magna*. Invading alien parasites not only threaten native individual hosts but also influence host populations, thus potentially also modifying parasite communities and interactions. The host range of *F. magna* includes a variety of potential hosts, notably other Lymnaeidae as potential intermediate hosts and various ungulates, including sheep and cattle, as final hosts. Because eradication after medical treatment was unsuccessful, and due to the risk of further spread of the parasite into unaffected regions, enhanced control strategies need to be developed. We recommend assessment of introduction pathways and dispersal, continuous monitoring of host abundance and distribution and the prevalence of flukes in intermediate and final hosts, as well as coordinated and concerted actions with neighbouring countries. This strategy could help to reduce potential negative impacts of this and other invasive parasites on host populations in Europe.

## Abstract (de)

Der Amerikanische Riesenleberegel *Fascioloides magna,* eine aus Nordamerika eingeschleppte Art, wurde in Österreich in freier Wildbahn erstmals im Jahr 2000 in den Donau-Auen östlich von Wien nachgewiesen. Seither wurden zahlreiche Daten über die Epidemiologie des Parasiten in den Schnecken-Zwischenwirten und in den Endwirten erhoben. Schnecken der Art *Galba truncatula* dienen als Zwischenwirte und Rothirsch, Reh und Damhirsch als Endwirte. *G. truncatula* ist im Gebiet häufig und vor allem an den schlammigen Ufern langsam fließender Gewässer zu finden. Prävalenzen im Rotwild (20–100 %) sind wesentlich höher als in den Zwischenwirten (0,03–0,2 %). Trotz medikamentöser Behandlung, eines Teils der betroffenen Wildpopulation hat sich der Parasit erfolgreich in den Auen beiderseits der Donau südöstlich von Wien etablieren können. Erste genetische Analysen weisen darauf hin, dass die Infektion der österreichischen Wildpopulationen ihren Ursprung in Tschechien oder in den östlich angrenzenden Populationen des Donau-Fluss-Systems hat. In den Leitha-Auen, die durch Wildbrücken mit den Karpaten und Donau-Auen verbunden werden, wurden die Parasiten bisher nicht festgestellt. Doch das Vorkommen geeigneter Zwischen- und Endwirte machen sie zu potentiellen Lebensräumen für die Riesenleberegel. Eingeschleppte/eingewanderte (Neobiota) Parasiten stellen nicht nur eine Bedrohung betroffener Wirtsindividuen und Populationen dar, sondern verändern auch bestehende Strukturen der Wirt-Parasiten-Gemeinschaft und deren Interaktionen. Das Wirtsspektrum von *F. magna* beinhaltet eine Reihe weiterer Arten, namentlich verschiedene Schlammschnecken als Zwischenwirte und diverse Huftiere einschließlich Rinder und Schafe als Endwirte. Weil die Medikation der Endwirte keine zufriedenstellenden Resultate erbrachte, und wegen des Risikos einer weiteren Ausbreitung des Parasiten, sollten wirksamen Strategien zur Kontrolle entwickelt werden. Wir empfehlen jedenfalls Untersuchungen zur Einschätzung der Mechanismen der Einschleppung und Ausbreitung, regelmäßiges Monitoring der Wirtsarten und des Vorkommens und der Prävalenz der Parasiten. Strategien und Maßnahmen sollten mit den benachbarten Ländern koordiniert werden. Diese Strategie könnte negative Auswirkungen der Parasiten auf die europäischen Populationen potentieller Wirtsorganismen reduzieren und wichtige Informationen über die Mechanismen der Einschleppung und Ausbreitung pathogener Neobiota vermitteln.

## Introduction

**Fig. 1:**
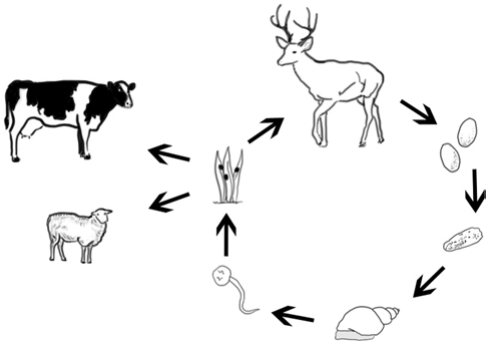
Life cycle of *Fascioloides magna*. (Designed by M. Haider)

**Fig. 2:**
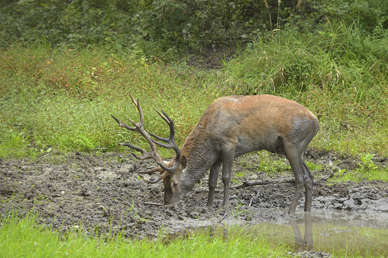
A red deer *Cervus elaphus*, the main final host in Austria, at a watering hole. (Photo: K. Kracher)

**Fig. 3:**
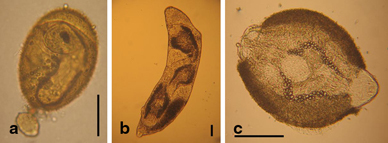
Egg and larval stages of *Fascioloides magna*: egg with opened operculum and miracidium inside (**a**), redia with cercariae inside (**b**) and encysting cercaria transforming into a metacercaria (**c**); scale bar = 50 µ. (Photos: Natural History Museum Vienna

**Fig. 4:**
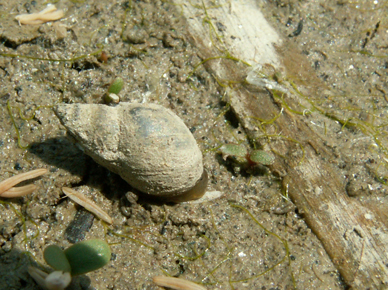
Lymnaeid snail *Galba truncatula* in the mud near a river shore; shell height: approximately 8 mm. (Photo: Natural History Museum Vienna)

**Fig. 5:**
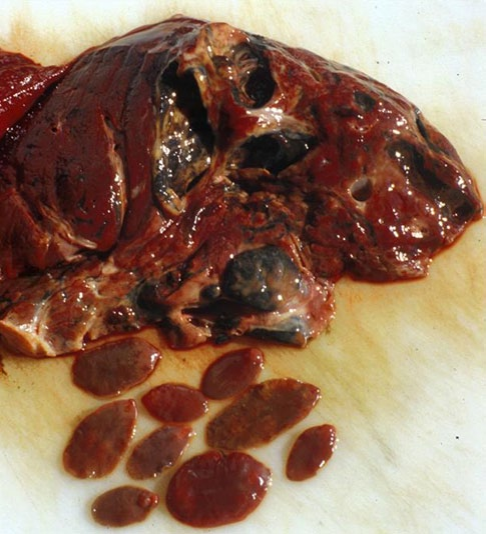
Liver with adult flukes of *Fascioloides magna*. (Photo: J. Ursprung)

The giant liver fluke *Fascioloides magna* (Bassi 1875) is a parasite in the liver of ungulates, preferentially cervids. Development of the sporocysts, rediae and cercariae takes place within lymnaeid snail hosts. Infection of final hosts occurs via ingestion of metacercariae, similar as in the European common liver fluke *Fasciola hepatica* (L) [1, 2] (life cycle, main hosts and larval stages see (Fig. 1, 2, 3, 4 and 5). *F. magna* proved to be moderately to severely pathogenic, depending on intensity of infection and host species [3–5]. Humans have not yet been shown to be susceptible to *F. magna*. The native distribution range of *F. magna* is North America [5]. However, *F. magna* was discovered and first described in Italy by Bassi [6], and it was only later determined that this species originates from North America [7, 8]. Further occurrences in various European countries within the twentieth and twenty-first centuries are documented [9–13]. Several persistent central European foci are known from sites in the Czech Republic since the late 1940s or even earlier [11–15]. More recent findings in Slovakia [16, 17], Hungary [18, 19], Croatia [20, 21], Serbia [22] and recently Germany [23] suggest that the parasite is still spreading. The dispersal downstream of the Danube River seems to originate from well-established and stable populations in the Czech Republic, although repeated introductions via introduced and infected hosts must also be assumed [24]. In Austria, the species was first documented in the wild in the Danube floodplains east of Vienna in 2000 [25]. Since then, several studies have described the epidemiology, pathology and ecological hot spots of infection of this Austrian population.

The aims of this article are

- to review the state of research in Austria and to summarize the perspectives raised in past studies,
- to assess the origin of the Austrian population by comparing available sequence data,
- to discuss possible control measures,
- to present first results of a parasitological screening in habitats close to the infested areas with regard to the incidence of fasciolids and
- to discuss the risks of spreading via natural migration and anthropogenic transportation of hosts and parasites.

## Occurrence and epidemiology of *F. magna* in Austria

First records of *F. magna* in Austria stem from 1982 in animals from a game husbandry in Lower Austria [26].

The author detected the flukes in one fallow deer (*Dama dama*) individual that had been imported from the Netherlands via a game husbandry in Upper Austria. Clearly, the parasites had been introduced together with the final host. Eggs of fasciolid appearance were found in the faeces of some other host individuals, but not further specified. There was no indication that the infection had escaped from this enclosure.

Almost 20 years later, the next record of *F. magna* in Austria originated from red deer (*Cervus elaphus*) and roe deer (*Capreolus capreolus*) from a hunting area at Fischamend in the floodplains at the southern bank of the Danube River east of Vienna. This was the first record in the wild for Austria [25]. The parasite, which originally was introduced to Europe with American deer (e.g. wapiti, white tailed deer), is suspected to have colonized Austrian deer populations via Hungary and Slovakia, both countries harbouring infested populations in the Danube floodplains nearby. These parasites are supposed to originate from populations that have inhabited different localities in the Czech Republic for decades [13, 24]. Due to the high prevalence in the Austrian red deer population (up to 100 %) and several cases of perished roe deer most likely caused by *F. magna* infection, medical treatment with triclabendazole was initiated at the feeding places in this particular area [27, 28]. This treatment continues, in a modified application method, until today [29, 30]. The Hunters Association of Lower Austria and the Austrian Federal Forests have initiated several studies to observe the distribution and dispersal of the parasite in the region. Since 2001, livers of killed or perished deer are being sent to a veterinarian for examination, and the results have been summarized annually and published hitherto twice [28, 29]. These reports provided evidence that medication was insufficient to eradicate the parasite. Prevalence decreased reasonably well within the first 6 years, but increased again in 2006. However, the drop in the intensities over the years was clearly due to medication. This trend is confirmed for 2011 and 2012. Moreover, in 2012, the first occurrence of *F. magna* in fallow deer (*D. dama*) was recorded in Austria in the wild [30]. Models considering habitat availability along with the abundances of deer and intermediate host snails were developed [31] to assess infection risks in the region. The authorities of the National Park Donau-Auen, which administer large parts of the floodplains between Vienna and Bratislava, have commissioned, together with the hunters association, studies within the floodplains along both sides of the Danube and at the lowest section of March River from August 2004 to September 2005 [32, 33]. Special attention was given to the distribution of intermediate host snails, particularly *Galba truncatula*, and its infection with fasciolids. These studies demonstrated a very low prevalence of *F. magna* (0.03 %) and even lower of *F. hepatica* (0.01 %) in more than 10,000 snails investigated [33]. Within the same period, the infection of deer was relatively low (30 % in 2004, 13 % in 2005) [29].

Monitoring of the high-risk areas near Orth at the northern banks of the Danube, commissioned by the Austrian Federal Forests, showed that the parasite cycle is well established in snails and deer in this area, as it was in other parts of the region in 2008–2009. Faeces of red deer and more than 3,000 *G. truncatula* snails were investigated [34, 35]. Prevalences in snails were still low (0.3 %), but a magnitude higher than in the study from Hörweg et al. [33]. These data fit well to increased prevalences in deer from 2006 to 2009 (ranging from 72.7 to 40 %) despite continuous medication in part of the region [29].

The density of *G. truncatula* snails was highest at periodically flooded areas along swampy shorelines of running waters. The seasonal shell size distribution implied a bimodal reproduction cycle [33, 34]. Moreover, frequent findings of *F. magna* and *F. hepatica* rediae in July and August point to seasonality of snail infections [32, 34].

Considering that the development of *F. magna* takes approximately 6–7 weeks in its snail host [10, 32], a high level of cercarial shedding in late summer/autumn is likely. This finding agrees with previous reports about *F. hepatica* showing the highest infection risk for final hosts in late summer and autumn in Europe [1, 36]. Also in agreement with previous studies [37, 38], snails with larger shell heights showed an increased prevalence of digenean trematode infections. For example, in the study of Haider et al. [34], the prevalence of trematode infections in snails with shell heights > 6 mm was 4.69 % (*n* = 384) compared with smaller individuals with only 2.12 % (*n* = 3,060); Sattmann and Hörweg [32] found 68.03 % of the total trematode infections in snails with > 5 mm shell height (*n* = 10,059).

## On the origin of *F. magna* populations in Austria

The description of *F. magna* was based on material from its first record in Europe [6] from a game reserve near Turino, Italy. It was again Bassi [7] who indicated that *F. magna* originated from North America. Within the following decades, *F. magna* was reported from several European countries, and Kralová-Hromadova et al. [24], based on genetic data, demonstrated that European populations have multiple origins and that the populations in the Czech Republic are of a different origin than those from Italy. Czech populations originate from at least five clades descending from different regions of North America. The authors argue that flukes from Slovakia, Hungary and Croatia apparently stem from two of those Czech clades and that these clades are now dispersing Danube downstream. The authors did not include flukes from the Austrian populations in their comparative studies. We therefore compared the gene sequences of Austrian *F. magna* with data from the literature/gene bank. Two Austrian *F. magna* isolates could be identified below the species level according to the haplotype system based on sequence data of the cytochrome c oxidase subunit I (*cox1*) and the nicotinamide dehydrogenase subunit I (*nad1*), respectively [24, 39]. Strain FmA_R was an adult worm isolated from a red deer from Fischamend, southern shore of the Danube; strain FmA _86 was a redia isolated from a *Galba truncatula* from the locality Entenhaufen (near Orth), northern shore of the Danube. From both isolates, the complete sequences for both the *cox1* and *nad1* genes were obtained. In the *cox1* gene, both isolates, FmA_R and FmA_86, are 439 bp long and show 100 % sequence identity to each other, both showing also 100 % sequence identity (439/439 bp) to the haplotype 3 isolates from the Czech Republic (GU599864), Slovakia (GU599865), Hungary (GU599866) and Croatia (GU599867). They show the next highest sequence identities (435/439 bp) to haplotype 1, again with equal identities to the isolates from Italy, Canada and USA (GU599860, GU599861 and GU599862, respectively).

**Fig. 6:**
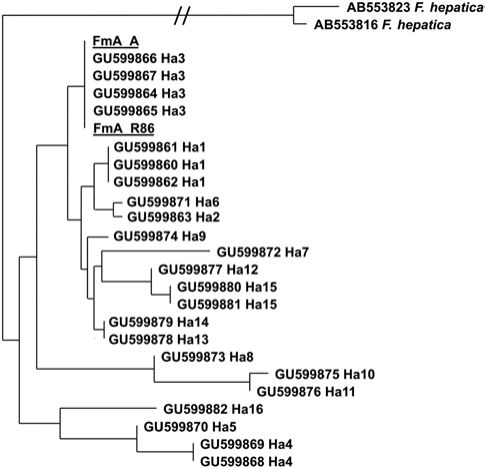
Cluster analysis of the *COX1* gene using two strains of *Fasciola hepatica* (AB553816 and AB553823) as an outgroup. Note that branches partially have been truncated for reasons of space (indicated by *double slash*). Detailed explanation is provided in the section ‘On the origin of *F. magna* populations in Austria’

For visualization, a cluster analysis was performed (Fig. 6). In brief, the obtained sequences were compared with reference sequences from GenBank by subsequent pairwise alignment using CLUSTAL X [40]. The resulting alignment was edited using GENEDOC [41]; primer sites were excluded from the alignment. Cluster analysis was performed using the PHYLIP 3.63 package using neighbour joining and maximum likelihood as evolutionary models and generating 100 bootstrap replicates. A consensus trees was obtained from the resulting trees using CONSENSE and prepared as figure with the TREEVIEW application. The tree was rooted with two sequences of *F. hepatica* (AB553816 and AB553823).

In the *nad1* gene, FmA_R and FmA_86 both gave a 405-bp-long sequence, with 100 % sequence identity to each other and also to the haplotype 4 isolates from the Czech Republic (GU599837) and Slovakia (GU599841).The next highest identity (404/405 bp) was found with haplotype 6 from the Czech Republic (GU599839) and with haplotype 16 (402/405) from the USA (GU599856/ GU599858). Sequence identity to haplotype 3 was 400/405 bp with the isolates from Italy and Canada (GU599836/GU599845).

Sequence data obtained for the current work were deposited in GenBank and are available under the following accession numbers: KF784787–KF784790.

## *F. magna:* an alien species

*F. magna* was introduced to Europe by human activities and reproduces in the wild—it must therefore be considered an alien species [27, 42, 43]. Biological invasions—the occurrence and spread of species beyond their natural range—are an increasingly important element of global change [44] and considered a major threat to biodiversity [45–47]. Organisms are continuously translocated into new environments by natural forces and, increasingly, by human activities [44, 48]. Parasitic invaders may arrive together with their hosts and may infest native host species, causing exceptionally negative impacts in their new habitats (e.g. eel swim bladder nematode, crayfish plague, *Varroa destructor*). Due to missing co-evolutionary adaptations, alien parasites may affect their new hosts more seriously than their native hosts. Thus, they may severely impact not only the individual host but also significantly change the host population dynamics [49]. Invasive parasites may also alter the natural parasite communities because they are forced to establish new niches within the existing communities. The expectation is that successful establishment of a new parasite alters the parasite community structure of the respective native hosts*. F. magna*, in North America, uses a number of lymnaeid snails as intermediate hosts. In Europe, it has been reported from lymnaeids, particularly from *G. truncatula*, which is also the main intermediate host for *F. hepatica*, the European liver fluke. Further native snails of the genera* Stagnicola* and *Radix* have been confirmed as potential intermediate hosts in Europe [11, 12, 50–52]. Also, introduced snail species such as *Pseudosuccinea* spp. may potentially provide host reservoirs [53]. Native red deer proved to be suitable as final hosts for reproduction of *F. magna* in Europe, but other native and introduced cervids (e.g. *C. capreolus, D. dama, Odocoileus virginianus*) and ungulates (cattle, sheep) have been reported as hosts as well. This calls for studying the competitive effects of *F. magna* within the parasite communities of intermediate and final hosts to learn more about its impact on other parasites, e.g. on economically and medically important species such as *F. hepatica* and *Paramphistomum* sp*.*: competitive effects could play a role as biological control mechanisms [54–56]. It should also be noted that climatic changes may alter the population dynamics of hosts and parasites as well [57–63].

## Control of alien parasite species

Invasive species are defined as alien species the introduction and/or spread of which may threaten biological diversity or have other unforeseen consequences, i.e. having negative economic or health impacts [64]. A comprehensive legal instrument to tackle invasive alien species at the European Union (EU) level is part of the EU Biodiversity Strategy 2020 and expected to be published in 2014 [65]. In addition, member state action may be necessary to reduce possible negative impacts at the regional or local level. Cooperation with neighbouring countries should be encouraged to concentrate control actions. Policy papers on invasive alien species often do not specifically refer to parasites or specify their effects, despite numerous documented examples with dramatic impacts [66, 67].

In Austria, the legally not-binding Action Plan on Invasive Alien Species [68] suggests measures and provides information on responsibilities related to Invasive Alien Species (IAS). It further includes a list of those invasive alien species that negatively affect biodiversity based on expert opinion; it does not include *F. magna*, although the species is considered to be of economic and animal health concern. Intentional release of living animals in Austria is regulated by nine different regional state laws on nature conservation, hunting and fishing and is also touched by federal laws on animal trade, on animal welfare, animal diseases, regulations on keeping animals and others [65].

Whereas some introduced vertebrates and invertebrates have received particular attention as threats to biodiversity, and some arthropods and molluscs were perceived as pests or vectors of infectious diseases [69], other invertebrates have been largely neglected by research and reporting. Monitoring of the abundance and spreading as well as further scientific research on parasite dispersal routes and impacts should be reinforced in environments that are already infested and in regions at risk of colonization. Regarding control, no legal demand is in place, and the results of medical treatment were unsatisfactory in the respective environments along the southern shore of Danube [29, 34]. As the National Park Donau-Auen covers big parts of the area settled by the parasite, in these protected zones, medical treatment was not accepted, based on conservation arguments.

Alternative control measures to govern the habitation, abundance, activity and migration of deer should be considered. Parasite monitoring is essential to maintain preparedness should further actions be needed.

## Parasite–host screening in surrounding areas

An ongoing study initiated by the Hunters Association of Lower Austria and the Hunters Society ‘Grünes Kreuz’ is designed to clarify the risks of spreading of the infection. This is particularly relevant due to upcoming measures to promote the genetic exchange of deer populations by bridging highways to connect isolated host populations. In the course of screening freshwater snails and digenean stages along the Leitha river system (between the villages Götzendorf and Potzneusiedl), approximately 1,300 lymnaeid snails have been investigated. No fasciolids, but several other trematodes (Schistosomatidae, Echinostomatidae, etc.), were found.

The abundance of *G. truncatula* was lower than in Danube backwaters [33, 34], but snails were still abundant at some localities on the Leitha River. Other suitable hosts, such as *Radix* spp., were rarely found, and *Stagnicola* spp. was not recorded at all. Concerning final hosts, only small red deer populations occur in the investigated areas (personal communication of local hunters), whereas roe deer is abundant. Red deer (and fallow deer) and roe deer are common European definite hosts, but roe deer seems to be an aberrant non-specific host in which migration of juveniles usually has a lethal effect [5]. However, worms may mature occasionally [11]. Several ungulates are known to be aberrant or dead-end hosts, like cattle, horse, sheep, goats, pigs and others [12, 70], the epidemiological meaning of which should be still scrutinized. Nevertheless, the abundance of intermediate and final hosts in this region provides the potential of parasite transmission [71]. Especially with the planned facilitation of the dispersal and exchange of red deer populations (see the next paragraph), the parasite may establish in the Leitha environments and other areas along red deer migration routes, e.g. to the Alps via the Leitha Mountains or to Hungary.

## *F. magna:* the risks of spreading

Dispersal of parasite species is caused by dispersal of their hosts and/or by dispersal of free-living stages. *F. magna* might be carried with deer by natural migration or by transportation through humans. Intermediate host snails may disseminate parasite stages by active movement only over short distances in the water. Passive transportation, however, by water, strong winds, animals and human activities is more likely for small amphibious snails like *G. truncatula* because they can easily survive lengthier harsh conditions such as droughts and floods. Such potential transportation includes flooding, adhesion in hairs of mammals and feathers of birds as well as transportation of soil and plants for commercial reasons. Free stages of the parasite (eggs, miracidia, cercariae, metacercaria) might be transported by water and rarely by wind (e.g. with vegetation). Therefore, it is unsurprising that *F. magna* apparently disperses downstream along the Danube River system. Other dispersal routes must be considered as well, especially along natural migrations routes of game animals. Most relevant in this regard is the Alpine-Carpathian-Corridor project, designed to re-establish former natural migration routes for wildlife (http://www.alpenkarpatenkorridor.at/). The ecological benefits of such corridors of connected habitats are unquestioned. Nonetheless, hunters and conservation biologists have to consider possible disadvantages of opening migration routes and must be aware of the infection risks. In this potential scenario, the invasion of Austrian alpine regions by *F. magna* needs to be considered and monitored. Moreover, besides the possible negative effects of parasites, also beneficial implications should be considered. One scenario is that extant game populations with high genetic diversity might better resist parasite pressure [72] and that efficient migration corridors provide scope for climate change adaptation [73].

## Discussion

*F*. *magna* is an alien species in Europe. In Austria and neighbouring countries, this liver fluke has been reported for several years, particularly in red deer and roe deer. It has a similar life cycle and ecology of transmission as the closely related European liver fluke. As several species of ungulates are potential final hosts and as one suitable intermediate host, *G. truncatula*, is widely distributed and abundant in the entire region, we expect further spread of the parasite in the future. Monitoring and research projects have shown high variability in prevalence and intensity in final and intermediate hosts. This might on the one hand reflect population fluctuations in snails caused by environmental dynamics, which is typical for floodplain forests [74, 75], and on the other hand reflect activity patterns of deer. Conspicuous differences in the prevalences in snails and deer may be explained by differences in activity patterns, densities and life cycles of the respective host populations and of parasite stages. *G. truncatula* lives up to 2 years, with a few weeks’ residence time of parasite stages. Deer survive up to 20 years, and adult liver flukes may thrive several years within the final host. Furthermore, the transmission via metacercariae to deer might be more efficient than via miracidia to snails because the survival time of metacercariae is much longer [76, 77]. Few indications on seasonal peaks of cercarial hatching in summer fit well with data from literature [1, 36]. Data of deer infection show that medication obviously has decreased intensity but did not affect the prevalence persistently [29, 34]. Snail screening in environments of the Leitha Auen, south of the affected area at the Austrian Danube, has yielded no *F. magna* infection, but the environmental conditions and availability of hosts underline the colonization potential [71].

This study shows that *F. magna* from two Austrian samples (one from a snail and one from a red deer) apparently is closely related to neighbouring populations of Danube in Slovakia, Hungary or Czech Republic. Thus, our data indicate that *F. magna* invaded to the Austrian habitats from Slovakia and/or Hungary because these are the geographically closest areas and they shelter the parasite for some time longer than Austria [16, 18]. This supports the hypothesis of Králová-Hromadová et al. [24] that the giant liver fluke spreads along the Danube system originating from populations in the Czech Republic, where the cycle has been established for decades in several localities.

As *F. magna* is a parasite in a variety of native and alien hosts in Europe, the newcomer may well alter the parasite communities in intermediate and final hosts [54, 56, 78, 79]. *F. magna* may influence population dynamics of host and parasites and affect biodiversity of these communities. In this respect, alien parasites in general and *F. magna* in particular may become important by means of wildlife conservation. *F. magna* might also serve as model organism to gain more information on the mechanisms and strategies of invasive alien parasites and on parasite dispersal in nature in general. This concerns human parasites, e.g. *F. hepatica*, endemic to Eurasia but invasive in all other continents [58, 67], and other parasites of ungulates with similar life history, e.g. *Paramphistomum* spp. It should also be noted that *F. magna* has a broad host range, including various native and introduced snails, and has a high potential to infect different cervids and other ungulates, including domestic sheep and cattle [5, 11, 13, 80] and even wild boar [23, 81]. This has serious implications for animal health. No strategy for the control of this parasite exists in Europe, and we strongly recommend the development of a coordinated European Action Plan to reduce potential negative impacts of this and other invasive parasites in Europe.
